# Protecting mothers and their infants through screening and comprehensive management of sexually transmitted infections in antenatal care in Zimbabwe (PROMISE): study protocol for an open label individually randomised controlled trial

**DOI:** 10.1136/bmjopen-2026-126249

**Published:** 2026-10-15

**Authors:** Leyla Larsson, Jacqueline Kabongo, Grant Murewanhema, Victoria Simms, Ethel Dauya, Tsitsi Bandason, Constance R S Mackworth-Young, Fiammetta Maria Bozzani, Robert Butcher, Anna Machiha, Nicola Low, Jonathan P Sturgeon, Hannah Blencowe, Chido Dziva Chikwari, Lynda Stranix-Chibanda, Agnes Juru, Lucia Sisya, Sarah Bernays, Katharina Kranzer, Michael Marks, Rashida A Ferrand, PROMISE trial consortium

**Affiliations:** 1 Department of Clinical Research, London School of Hygiene & Tropical Medicine, London, UK; 2 The Health Research Unit Zimbabwe, Biomedical Research and Training Institute, Harare, Zimbabwe; 3 Faculty of Medicine and Health Sciences, University of Zimbabwe, Harare, Zimbabwe; 4 Department of Infectious Disease Epidemiology, London School of Hygiene & Tropical Medicine, London, UK; 5 Department of Global Health and Development, London School of Hygiene & Tropical Medicine, London, UK; 6 AIDS and TB Unit, Ministry of Health and Child Welfare, Harare, Harare Province, Zimbabwe; 7 Institute of Social and Preventive Medicine, University of Bern, Bern, Switzerland; 8 Section of Paediatric Infectious Diseases, Imperial College London, London, UK; 9 Clinical Trials Research Centre, University of Zimbabwe Faculty of Medicine and Health Sciences, Harare, Zimbabwe; 10 National Microbiology Reference Laboratory, Harare, Zimbabwe; 11 School of Public Health, University of Sydney, Sydney, New South Wales, Australia; 12 Institute of Infectious Diseases and Tropical Medicine, Ludwig Maximilian University of Munich Faculty of Medicine, Munich, BY, Germany

**Keywords:** Pregnancy, Sexually Transmitted Disease, Africa, Clinical Trial, Randomized Controlled Trial, Maternal medicine

## Abstract

**Introduction:**

Observational studies suggest that sexually transmitted infections (STIs) during pregnancy are associated with a range of adverse outcomes for both mothers and babies, including preterm birth, low birth weight, and stillbirth.

Furthermore, studies have shown a higher prevalence of STIs among those living with HIV. In low-resource settings, syndromic STI management is the standard approach which has low sensitivity as more than 80% of curable STIs are asymptomatic. The PROMISE trial evaluates whether screening and comprehensive management of three curable STIs, *Chlamydia trachomatis*, *Neisseria gonorrhoeae* and *Trichomonas vaginalis*, integrated into standard antenatal care can reduce adverse maternal and neonatal outcomes in a high HIV/STI prevalence setting.

**Methods and analysis:**

PROMISE is an individually randomised controlled trial conducted in 14 antenatal clinics in Harare, Zimbabwe. Women are eligible if at <24 weeks gestation, confirmed by ultrasound. Women in the intervention arm receive screening for the three STIs two times during pregnancy (at <24 and at 32–34 weeks’ gestation), with treatment and partner management for those with positive test results. Women in the control arm and symptomatic women in the intervention arm receive standard of care (syndromic management). The primary outcome is a composite of preterm birth (<37 weeks’ gestation), low birth weight (<2500 g), and stillbirth (born ≥22 weeks without sign of life). Secondary outcomes include individual components of the composite outcome, prelabour rupture of membranes, babies that are small for gestational age, neonatal death or conjunctivitis in babies within the first 6 weeks of birth. The planned sample size is 12 780 pregnant women (6390 per arm). A cost-effectiveness analysis will estimate the incremental cost per disability-adjusted life year averted. A process evaluation will explore acceptability, feasibility, contextual influences and system-level considerations for scale-up.

**Ethics and dissemination:**

Ethical approval for the trial has been granted by the Medical Research Council of Zimbabwe, the Biomedical Research and Training Institute Institutional Review Board, the Joint Research and Ethics Committee, the Sally Mugabe Central Hospital Ethics Committee and the London School of Hygiene & Tropical Medicine Research Ethics Committee. Findings will be disseminated through peer-reviewed publications, conference presentations, engagement with national and international policymakers and community dissemination workshops co-developed with the trial’s Community Advisory Board.

**Trial registration number:**

PACTR202505577399427 (Pan-African Clinical Trials Registry).

**STRENGTHS AND LIMITATIONS OF THIS STUDY**

- This is a large individually randomised trial powered for each individual outcome of the composite primary outcome that, unlike previous sexually transmitted infection (STI) screening trials in low-resource settings, includes stillbirth alongside preterm birth and low birth weight in its primary composite outcome.
- The intervention is embedded within routine antenatal care across 14 public sector clinics in a high HIV/STI prevalence urban setting, so trial procedures and findings should be directly relevant to comparable low-resource settings where syndromic management remains the standard of care.
- Gestational age at birth is ascertained using a validated artificial intelligence-supported ultrasound tool, standardising a key outcome measure across a multi-clinic recruitment network.
- Because the trial evaluates an antenatal care pathway rather than a drug or device, participants and clinical staff cannot be blinded to allocation, although outcome analyses will be conducted by a statistician blinded to the trial arm.
- Deferred disclosure and treatment of STI results in the control arm, required to preserve a valid comparison with standard syndromic care, means some women with an infection remain untreated until after birth (if asymptomatic); this trade-off has been approved by the trial’s ethics committees and is mitigated through safety monitoring by an independent Data Safety and Monitoring Board.

## Introduction

### Background and rationale

 By 2021, none of the global targets set by the WHO to achieve control of curable sexually transmitted infections (STIs) have been met.^1^ In 2021, WHO estimated that approximately 374 million people acquired one of four curable STIs globally: 128 million for chlamydia (caused by *Chlamydia trachomatis* (Ct)), 82 million for gonorrhoea (caused by *Neisseria gonorrhoeae* (Ng)), 156 million for trichomoniasis (caused by *Trichomonas vaginalis* (Tv)) and 7 million for syphilis (caused by *Treponema pallidum* (Tp)) each year—equivalent to more than 1 million new infections every day.^1^ The global prevalence of some STIs is also increasing, particularly for multi-drug resistant gonorrhoea and syphilis.^2^ While regional estimates of STI prevalence among pregnant women vary, these four curable STIs remain common among pregnant women, particularly in Southern Africa, where a systematic review of studies conducted between 2015 and 2020 reported prevalences of Tv and Ct at 15.0%, Tp at 3.0% and Ng at 4.0%.^3^ Factors contributing to the inadequate control of STI and the failure to meet the global targets include insufficient health financing, limited global interest, and weak political commitment. Complex sociocultural barriers such as stigma, limited sexual health education and low condom use further undermine programme effectiveness and contribute to the persistent high prevalence of STIs.^4^

Observational studies suggest that STIs during pregnancy are associated with a range of adverse neonatal and maternal health outcomes.^5^ These include but are not limited to low birth weight (LBW), preterm delivery, stillbirths and early neonatal mortality.^6^ STIs during pregnancy are also associated with postnatal morbidity, causing ocular infections, sepsis or lower respiratory tract infections in infants.^7^ These are outcomes of immense public health importance. Globally, there are an estimated 2.6 million neonatal deaths and 1.9 million stillbirths every year (98% in low resource settings).^8^ Furthermore, babies born preterm, with LBW or are small for gestational age who survive the neonatal period remain at heightened risk of poor growth and development and death.^9^ These consequences contribute to an intergenerational cycle whereby poor growth in girls leads to short adult stature, increasing the risk of intrauterine growth retardation and obstetric complications.^10 11^

For curable STIs, in most low resource settings, the WHO-endorsed syndromic management approach is used, which has low sensitivity: more than 80% of curable STIs are asymptomatic and therefore remain untreated.^12 13^ Syndromic management also lacks specificity, leading to unnecessary exposure to broad spectrum antibiotics and potentially accelerating antimicrobial resistance (AMR), particularly for Ng.^14^

In recent years, the development of diagnostic platforms that are sensitive, specific, rapid and independent of sophisticated infrastructure has created an opportunity to transform STI management in low- and middle-income countries (LMICs) by moving away from syndromic management to management informed by aetiological testing.^15^ There is limited evidence on how best STI diagnostics should be deployed to achieve maximal public health impact. Recognising this gap, the WHO has called for evidence to guide the transition from syndromic management to diagnostic based approaches,^1^ which is likely to be central to reducing STI prevalence in LMICs.

One application of diagnostics is for screening for STIs in pregnancy. The rationale is the likely impact on maternal and child health outcomes and the opportunity to integrate screening within the WHO-recommended triple elimination programme of mother-to-child transmission of syphilis, HIV and hepatitis B.^15 16^ The WANTAIM (Papua New Guinea (PNG))^17^ and Philani Ndiphile (South Africa) trials are the only completed trials to have investigated the impact of STI screening on neonatal outcomes. Neither found an effect on the primary outcome (composite of LBW/prematurity).^18^

Within the PROMISE trial, we will investigate the effectiveness of STI screening and treatment for women during pregnancy with the aim of reducing poor maternal, neonatal and infant outcomes. Our trial differs in four key respects: (1) WANTAIM was conducted in PNGwhich is malaria endemic and where sulfadoxine-pyrimethamine prophylaxis was given to all women which may treat Ct, likely diluting the effect of the intervention^19 20^; (2) PNG is a low HIV prevalence setting; (3) our trial is powered to detect a smaller effect size compared with both trials and (4) our trial includes stillbirth in its composite outcome.

## Methods and analysis

### Objectives

PROMISE aims to investigate the impact of screening and comprehensive management of STIs within antenatal care on health outcomes in a high HIV/STI prevalence setting in Africa. Specific objectives are to investigate:

1. The impact of screening and comprehensive management of STIs within antenatal care on adverse maternal and neonatal outcomes.
2. The cost-effectiveness of the intervention.
3. The community needs and health system requirements to maximise acceptability, uptake and implementation success at scale.

The trial will also serve as a platform for multiple sub studies investigating the vaginal microbiome and inflammation due to STIs, the neonatal sequelae of STIs during pregnancy (ie, neonatal conjunctivitis and congenital syphilis), the effects of climate change (through heat and food/water insecurity) on maternal and birth outcomes, the epidemiology of hypertensive disorders of pregnancy and use of artificial intelligence-supported ultrasound to monitor foetal growth restriction.

### Trial design and study setting

PROMISE is an open label individually randomised parallel group two-arm superiority trial with a 1:1 allocation ratio. The trial will be conducted in Harare, the capital of Zimbabwe, where pregnant women accessing antenatal care and intending to deliver in Harare will be recruited from 14 antenatal clinics which were chosen owing to their high volume of women registering for antenatal care (figure 1). All chosen antenatal clinics are situated within primary healthcare settings in predominantly urban and peri-urban areas. Most clinics (n=12/14) participating in PROMISE also have delivery units.

**Figure 1:**
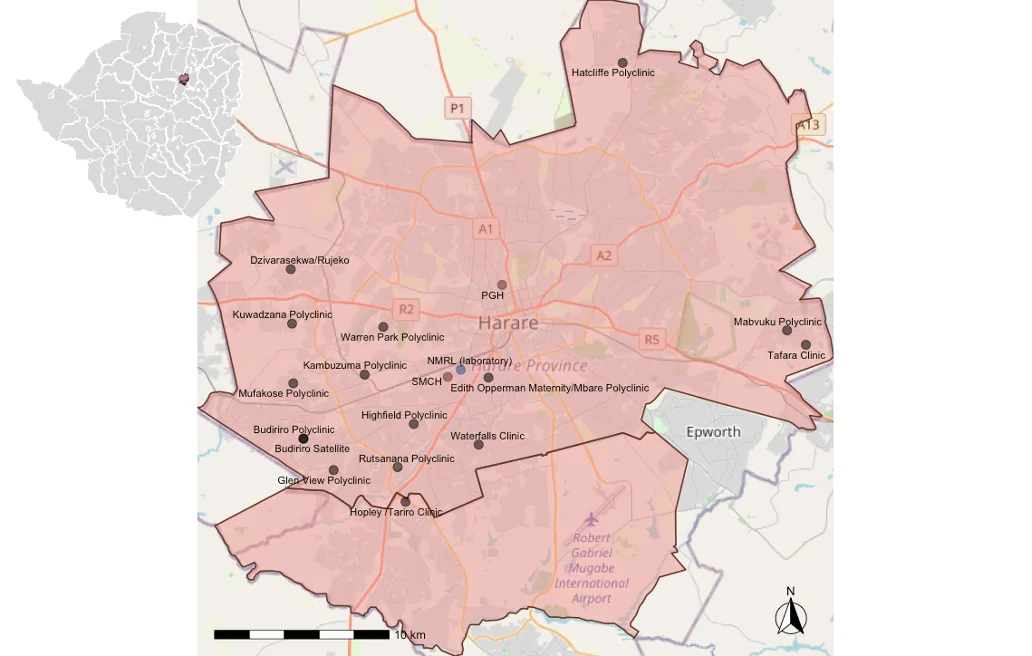
Map highlighting recruitment clinics, hospitals (brown) and laboratory (blue). NMRL, National Microbiology Reference Laboratory; PGH, Parirenyatwa Group of Hospitals; SMCH, Sally Mugabe Central Hospital.

There is evidence of the high prevalence of STIs among women registering for antenatal care in Harare and that deployment of rapid STI diagnostics in antenatal care is feasible and achievable in Zimbabwe.^21^ In the recently completed Investigating Point-of-care diagnostics for STIs and AMR in primary care in Zimbabwe study among 1001 pregnant women in Zimbabwe, uptake of STI testing was 91.1% among women who were offered screening and prevalence of curable STIs was high (Ct: 18.5%, Ng: 4.0%, Tv: 12.4%, any of Ct/Ng/Tv: 28.3%).^22^ Notably only 12% of STIs were symptomatic and would therefore not be identified by routine syndromic management.^22^

### Eligibility criteria

Inclusion criteria for pregnant women of any age,

- Minimum gestational age of 14 weeks and 0 days and maximum gestational age of 23 weeks and 6 days (ie, <24 weeks completed gestation) assessed through an artificial-intelligence supported ultrasound scan (FAMLI app).^23^
- Intend to be based in Harare for pregnancy, delivery and neonatal period.
- Agree to have vaginal swabs collected for STI testing.
- Able to provide reliable contact details to facilitate future participant tracing and postnatal follow-up.

Exclusion criteria include,

- Any condition that may prove fatal to the mother during the study period.
- Any other condition (social or medical) that, in the opinion of the study staff, would make study participation unsafe, including inability to provide consent.

Each antenatal clinic will have a study team comprising at least one research nurse and one research assistant, all of whom will be trained in study procedures, including obtaining informed consent. A member of the clinic study team will approach women at the antenatal clinic. Pregnant women will be assessed for eligibility to participate in the trial using the above-mentioned criteria (figure 2). If they meet the inclusion criteria, the pregnant women will be invited to watch an information video (https://www.youtube.com/watch?v=SRjOrwuExTQ) in the language of their choice (English or Shona (the local language)) on the study tablet followed by assessment of comprehension and electronic signature.

**Figure 2:**
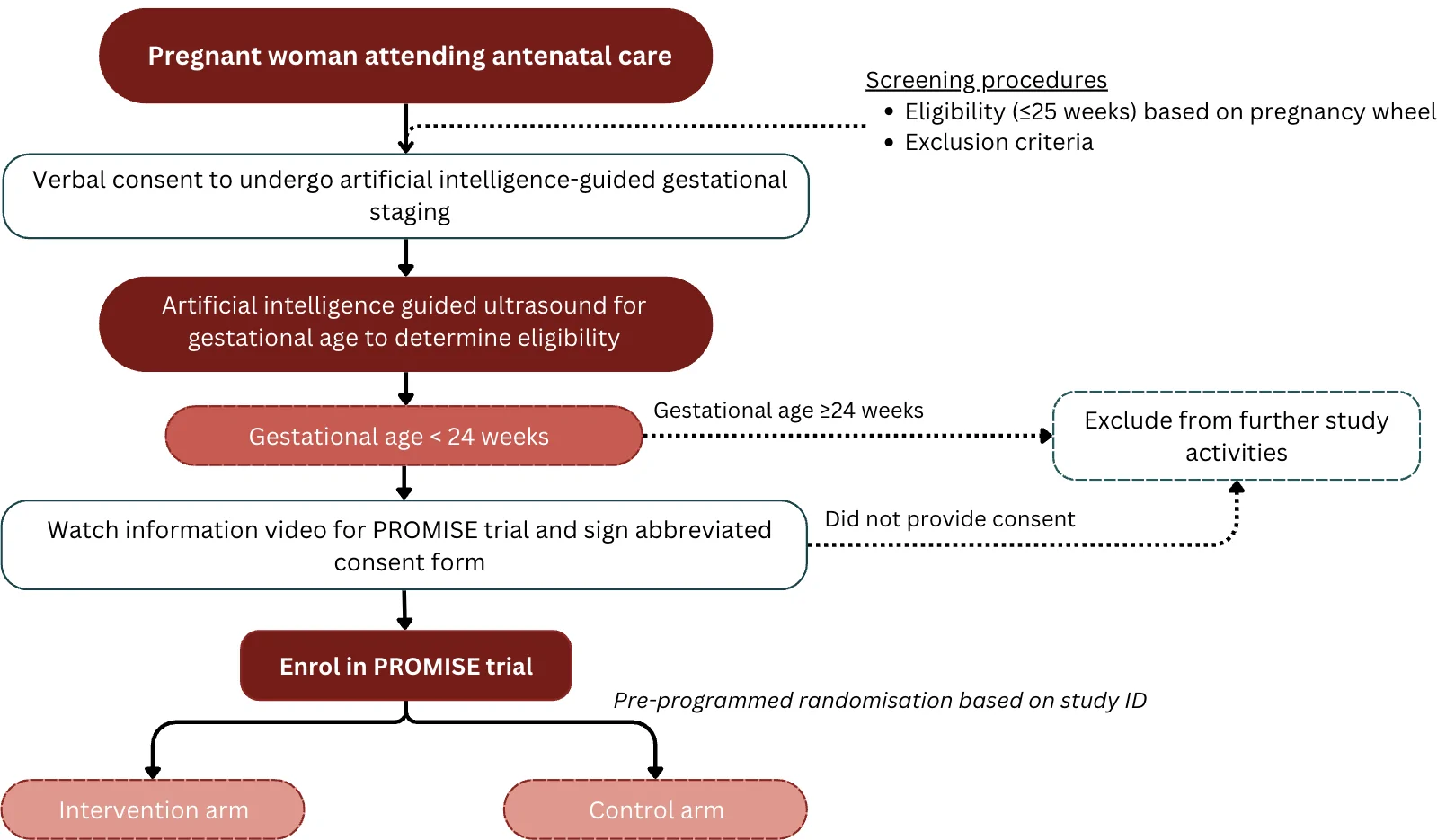
Screening and enrolment procedures in PROMISE *For sub studies.

### Additional consent provisions for collection and use of participant data and biological specimens

Beyond the main trial activities, PROMISE will also serve as a platform, supporting a suite of nested sub studies that will extend beyond evaluation of the main trial aims. These sub studies will span investigation of genital infection and inflammation, the vaginal microbiome, neonatal sequelae of maternal STIs, novel diagnostic approaches and environmental exposures, including heat exposure and food/water insecurity. By leveraging the core trial infrastructure, longitudinal follow-up and detailed clinical phenotyping of mothers and infants, PROMISE will enable mechanistic and contextual insights into pathways linking infection, inflammation and environmental stressors to adverse pregnancy and neonatal outcomes. Importantly, this design will strengthen the potential of PROMISE to generate evidence that will inform not only STI policy but also wider strategies to improve maternal and neonatal health in low resource settings. For each additional participation in sub studies (which could include collection of additional specimens), additional written consent will be sought by the research nurse. Separate protocols will be written for each sub study. Participants will be able to decline even if they consented to the participation in the trial.

### Intervention and comparator

Participants will be individually randomised to either the intervention or the control arm following confirmation of eligibility and informed consent. All study procedures will be embedded within routine antenatal services at participating clinics in Harare, Zimbabwe.

#### Intervention arm: screening and comprehensive management of STIs

The intervention is screening for three curable STIs (Ct, Ng, Tv), integrated within standard antenatal services. Screening will be conducted at two time points during pregnancy: at enrolment (<24 weeks’ gestation) and during the third trimester (32–34 weeks’ gestation with a 2-week buffer either side [ie, 30–36 weeks]). At each intervention study visit, a vaginal swab will be obtained by trained research nurses according to standard operating procedures. If a participant declines provider collection, the research nurse will offer instructions for self-collection. PROMISE will use a hub-and-spoke model for testing where samples will be tested for Ct, Ng and Tv using a CE-marked nucleic acid amplification test on the Roche cobas 6800 platform at the National Microbiology Reference Laboratory. Samples will be taken daily to the laboratory for storage until testing and test results are returned to the clinic within 1 week. Participants with a positive STI test result will be actively contacted via telephone by the study team and asked to return to the clinic for free treatment for each STI detected in accordance with Zimbabwe National STI Management Guidelines. Treatment will be administered by research nurses and will include antibiotics for the identified infection(s), risk reduction counselling and tailored partner notification support (ie, provision of expedited referral, participant referral or provider referral based on the participant’s needs).

#### Comparator arm: standard of care (syndromic management)

The comparator is syndromic management of STI symptoms, the current standard of care in Zimbabwe. In this approach, treatment will only be provided if participants have symptoms consistent with a syndrome (eg, vaginal discharge syndrome or pelvic inflammatory disease) during routine ANC visits according to national guidelines. For trial purposes, vaginal swabs will also be obtained from participants in the control arm at both visits, using the same procedures as in the intervention arm. However, the results will only be disclosed once the primary outcome has been ascertained. Participants with a positive result at the third trimester visit who have not already received syndromic treatment during pregnancy will be contacted and offered free treatment and comprehensive management as in the intervention arm in the early postnatal period (a positive result at enrolment which is negative in the third trimester will be assumed to have resolved).

##### Both arms

Routine ANC services, including clinical examinations, prophylactic treatments and referrals, will be delivered by clinic staff in both arms, with study personnel responsible only for trial-specific procedures and data collection. Participants who report symptoms suggestive of an STI at any visit will be managed syndromically at the time of presentation, regardless of trial arm, in line with standard of care. All participants in both arms also will receive routine opt-out HIV and syphilis testing at both visits. Syphilis diagnosis, staging, treatment and partner notification will be managed according to national protocols. Detailed descriptions of specimen collection, laboratory testing, treatment regimens, counselling and partner notification procedures are provided in the trial protocol and accompanying Standard Operating Procedures, which will be available from the investigators on request.

### Participant timeline

The participants will attend five study visits, all of which align with the standard antenatal visit schedule. Two visits will occur during pregnancy, where the intervention will be provided and vaginal swabs will be taken. Three visits will occur following delivery for outcome ascertainment. Depending on their involvement with sub studies in PROMISE, there may be additional visits for the mother and/or the infant (table 1).

**Table 1:** PROMISE trial schedule of events and study procedures at each visit for the main trial and sub studies

	Intervention visits		Outcome ascertainment		Sub-studies			
	Enrolment (<24 weeks)	Third trimester (32–34 weeks)	Birth (<72 hours after birth)	6 weeks	3 months	6 months	9 months	12 months
Main trial								
Screening	X							
AI ultrasound scan	X	X*						
Informed consent	X	X						
STI signs and symptoms	X	X						
Vaginal examination†	X†	X†						
Syndromic management‡	X‡	X‡						
Vaginal swab collection§	X	X						
Anthropometrics and vitals	X	X	X					
Study questionnaire	X	X	X	X				
HIV and syphilis testing	X	X						
STI treatment¶	X¶	X¶	X¶					
Eye symptom assessment			X	X				
Eye swab collection			X	X**				
Conjunctivitis treatment			X**	X**				
Sub-studies								
Vaginal swab (n=2, stored)††	X††	X††						
Food/water insecurity questionnaire (climate study)	X‡‡							
Blood samples (baby and mother)			X§§					
Corneal scarring assessment						X¶¶		
Blood sample for RPR testing			X***		X***	X***	X***	X***
Nasal swab collection			X†††					
Neurodevelopment								X‡‡‡

* An artificial intelligence ultrasound scan will be conducted in the third trimester but results merely stored and not used for clinical or study decision making.

† Only if the participant reports having symptoms related to STIs.

‡ Only if the participant reports having symptoms related to STIs and the vaginal examination indicates need for syndromic management. Relevant to both trial arms.

§ Swabs are collected from participants in both trial arms. Results are received immediately in the intervention arm and returned following primary outcome ascertainment in the control arm. Leftover eluate from testing is stored at −80 °C before shipment to the Wellcome Sanger Institute for metagenomic analyses.

¶ Provided in the intervention arm at both intervention visits only if positive and not previously treated syndromically. In the control arm, this is provided as soon as possible after outcome ascertainment at birth and only if not previously syndromically treated in the third trimester.

** Only if eye symptom assessments indicate necessary (at birth eye swabs are collected from all babies while at later visits only from symptomatic babies). Part of the sub study evaluating the epidemiology of neonatal conjunctivitis.

†† Only if consented to have additional samples collected for genital inflammation sub study which will undergo cytokine/proteomics analysis to characterise vaginal inflammation.

‡‡ Also includes a dietary diversity questionnaire. Temperature sensors are placed at 8 of the participating clinics and are monitoring temperature continuously throughout the study. Part of the climate change in pregnancy sub study.

§§ Blood samples collected to investigate the role of maternal inflammation and STI infection on the inflammation profile of the child. Samples will undergo biomarker analysis and transcriptomics at Imperial College London.

¶¶ All children who were diagnosed with neonatal conjunctivitis and who consent for follow-up will be assessed at 6 months for corneal scarring and compared with a selection of controls from the PROMISE cohort. Part of the sub study evaluating the epidemiology of neonatal conjunctivitis.

*** RPR testing on infants born to mothers who had syphilis in pregnancy. Part of the sub study evaluating the long term outcomes of babies exposed to syphilis in utero.

††† Nose swab will undergo PCR testing to test for carriage of *Treponema pallidum*. Part of the sub study evaluating the long term outcomes of babies exposed to syphilis in utero.

‡‡‡ Case control study among the children part of the congenital syphilis cohort. Will be compared with a random selection of children not exposed to syphilis in utero.

RPR, rapid plasma reagin; STI, sexually transmitted infection.

### Visits during pregnancy (intervention visits)

At enrolment, following screening and informed consent, the study team will provide a study card to the participant and will administer a questionnaire to collect information on age, socio-economic status, marital status, sexual history, family planning and obstetric history, previous STI diagnoses including HIV, presence of current STI symptoms and recent antibiotic use. They will then collect vaginal samples for STI testing and for storage (for sub studies if additional consent is provided) and anthropometrics (weight, height, blood pressure and mid upper arm circumference). Data regarding clinical examinations conducted by the antenatal clinic nurse will be extracted from the patient records. At the third trimester visit, pregnant women will (1) complete a follow-up questionnaire, (2) provide a vaginal sample for testing, (3) provide vaginal samples for storage and future use, (4) undergo a second ultrasound scan and (5) receive routine antenatal examinations appropriate for this stage of pregnancy. The second ultrasound scan will generate key data to support the development of additional AI modules on the FAMLI application.

### Visits following delivery (outcome ascertainment visits)

From the third trimester visit on, the study team will conduct weekly follow-up calls with participants until delivery. These calls will (1) monitor pregnancy status and anticipated delivery timing to ensure the birth visit can be scheduled within 72 hours of delivery and (2) serve as a reminder for participants to contact the study team immediately when labour begins. Trial outcomes will be ascertained during the follow-up visits conducted within 72 hours postnatally (birth visit) and at 6 weeks post-delivery. At the birth visit, the primary outcomes (composite of stillbirth, LBW and prematurity) will be assessed, while secondary outcomes will be evaluated at the 6-week visit (table 2).

**Table 2:** RE-AIM framework for PROMISE

*Element*	*Questions*	*Measures*	*Data sources/methods*
Reach (& acceptability)	What percent of eligible women received the intervention? What are the differences between those who received the intervention and those who opted out?	Recruitment rates Uptake of STI testing Uptake of STI treatment Uptake of partner notification (PN) and type of PN Socio-demographics of all eligible participants stratified by receiving intervention or those refusing	Enrolment tracking community engagement workshops
Effectiveness	What is the effect of the intervention on neonatal outcomes?	STI yield, prevalence and incidence Trial outcome	Study datasets
Adoption	What health system requirements are needed to adopt intervention at scale to overcome barriers and leverage facilitators?	Perceptions of policymakers, healthcare providers and pregnant women (and their significant others)	Stakeholder workshops, observational logs, semi-structured interviews
Implementation	What does the intervention cost? What support is required to sustain the intervention?	Cost-effectiveness data stakeholder perceptions of acceptability and readiness to deliver intervention at scale	Study datasets, stakeholder workshops, observational logs, semi-structured interviews
Maintenance	What resources will be needed for the intervention to be sustainable? What adaptations are needed to support integration into current service delivery?	Cost-effectiveness data Stakeholders' experience of intervention delivery, feasibility of modifications to maximise future implementation success	Study datasets, stakeholder workshops, observational logs, semi-structured interviews

RE-AIM, Reach, Effectiveness, Adoption, Implementation and Maintenance; STI, sexually transmitted infection.

All babies will have their eyes swabs taken at the birth visit to understand the prevalence of ocular pathogen carriage in newborns. The mother will be invited to attend the clinic for a PROMISE visit if the baby displays any indicative symptomatology of neonatal conjunctivitis (ie, eyes/eyelids displaying swelling and/or oedema, congestion of blood vessels or a purulent discharge) at any time between the birth visit and the 6-week visit. At this stage, a swab will be collected from each eye by the nurse.

### Outcomes

The primary trial outcome is a composite of the proportion of babies among total births born premature (before 37 completed weeks' gestation) and/or LBW (<2500 g) and/or stillbirths (born ≥22 weeks without sign of life).^24^ Gestational age at birth will be measured as the date of birth minus the gestational age at enrolment, assessed by artificial intelligence obstetric ultrasound scan.^23^ This measurement will be used to determine preterm birth and stillbirth. birth weight will be measured using study-specific calibrated scales, though the clinic-measured birth weight will also be recorded. The primary outcome will be measured at the maternal level for women with multiple pregnancies, that is, if any baby of a multiple pregnancy is preterm or of LBW, or stillborn, the mother will be recorded as having the outcome.

Secondary outcomes are proportions of: women with prelabour rupture of membranes (stratified by preterm or term), babies that are small for gestational age according to the INTERGROWTH-21 growth charts (among live births), babies who are premature (among live births), babies who are stillborn (among total births), babies with LBW (among live births), early and total neonatal deaths (0–7 and 0–28 days respectively; as a proportion of live births) and newborns with conjunctivitis within the first 6 weeks of birth (ascertained clinically at the birth and 6-week visits, as a proportion of live births).

### Process evaluation

A detailed mixed methods process evaluation will be conducted, guided by an implementation science focus and the Reach, Effectiveness, Adoption, Implementation and Maintenance framework (table 2).^25 26^ It will seek to understand the operational and structural factors required for scalability to provide guidance for sustainable implementation should it prove effective. Quantitative process data will be drawn from routine programme data collection and the study datasets. The qualitative process data will be collected at multiple time points during the trial through interviews with pregnant women and healthcare providers; community workshops with male partners, older women and traditional healers and birth attendants; biannual stakeholder meetings and ethnographic observations with pregnant women in their everyday lives. Throughout the trial, the process evaluation will remain flexible and dynamic to be responsive and adapt to emerging trial and process findings, and so additional data collection may be conducted to investigate emerging findings. Findings from the process evaluation which will provide insights into the implementation context will be recognised as valuable, regardless of the trial outcome.

### Cost-effectiveness and health economics

In parallel, PROMISE will include a cost-effectiveness analysis to assess the value for money of investing in integrated HIV and STI screening in ANC. The incremental costs and effects of the integrated model delivered in the intervention arm will be compared with the standard of care. Intervention delivery costs will be estimated from the healthcare provider’s perspective using a bottom-up (‘ingredients’) approach, where the economic costs of diagnosis and treatment inputs will be calculated to identify an average cost per woman screened. Costs from the patient perspective will not be included in the analysis. For the base case analysis, incremental cost-effectiveness ratios (ICERs) will be calculated for the trial outcomes. To maximise comparability with other trials, ICERs will also be presented in terms of cost per life-year saved and per disability-adjusted life-year (DALY) averted. If the intervention demonstrates clinical effectiveness, a cohort decision analytic model will be built, drawing on trial and available published data, to project costs and DALYs averted from the intervention over a newborn’s lifetime. Deterministic and probabilistic sensitivity analyses will be carried out to identify the parameters driving uncertainty in the cost-effectiveness estimates.

### Sample size

A 2010 review reported that Zimbabwe had a preterm birth rate of 17%, the fourth highest in the world.^27^ In 2015, the stillbirth rate was 30.5/1000 births, unchanged from 1980.^28^ The Sanitation, Hygiene, Infant Nutrition Efficacy (SHINE) trial, conducted in Zimbabwe, reported a combined 25% incidence of preterm birth, LBW and stillbirth in a sample of>5000 women.^29^ SHINE was however conducted in a rural area and these adverse outcomes may be lower in an urban area. We have estimated a 20% prevalence for PROMISE, a Harare-based trial. Prevalence of LBW in the 2015 Demographic Health Survey was 10% in both areas.^30^

Approximately 30% of women attending ANC are positive for any of Ct, Ng or Tv. We estimate that the prevalence of the composite outcome is 20% among women attending ANC. We assume a risk ratio (RR) of 2 for women with STIs, compared with those without STIs. These assumptions represent a composite outcome prevalence of 15.4% in women without STIs and 30.7% in women with STIs. We estimate that STI treatment could reduce the prevalence of the primary outcome by 15% overall or by 33% among women with an STI (ie, from 30.7% to 20.7%). Sample size was calculated using the following formula:


$$n_{1}={\left(Z_{1-\frac{\alpha }{2}}+\;Z_{1-\beta }\right)}^{2}\;\frac{p_{1}\;\left(1-\;p_{1}\right)+p_{2}\left(1-p_{2}\right)}{{\left(p_{1}-p_{2}\right)}^{2}}$$

With 5432 participants per arm (total 10 864), we would have 98% power to detect a 15% relative reduction in prevalence of the primary outcome in the intervention arm (ie, to 17%), assuming a prevalence of 20% in the control arm, with an alpha of 0.05. We aim to recruit 6390 women per arm (total 12 780) to allow for 15% loss to follow-up. The sample size has been calculated to give adequate statistical power to detect clinically meaningful reductions in each component of the primary outcome. For preterm birth, if 17% of women in the control arm give birth preterm, we have 90% power to detect a reduction of 2.4 percentage points to 14.7% prevalence in the intervention arm. If we estimate 10% prevalence of LBW in the control arm, we have 90% power to detect a reduction of 1.8%–8.2% in the intervention arm. The sample size of 5432 per arm would give 80% power to detect a 32.8% relative reduction in stillbirth prevalence (ie, to 1.48%) and 90% power to detect a 37.5% relative reduction to 1.38%.

Assuming a Ct prevalence of 18% (n=977 per arm) and a primary outcome prevalence of 30% among women with Ct, the sample size would give 90% power to detect a 21.6% relative reduction in the primary outcome (ie, to 23.5%). Among women with Ng (n=217 per arm), the study would have 80% power to detect a 38.3% relative reduction in prevalence from 30% to 18.5%.

### Randomisation

An independent statistician will generate the random allocation sequence using the *ralloc* command in Stata and securely send it directly to the data manager, who will programme it directly into tablet computers used for data collection at each study clinic. Stratified by clinic, block randomisation with varying block sizes will be used to allocate participants 1:1 to the intervention or control arm. Block length will vary between block sizes 2, 4, 6, 8, 10 distributed in the ratio 1:4:6:4:1. There will be no further stratification applied. Allocation will be concealed from staff and potential participants until after the participant has consented and been allocated a study ID. Once the participant’s screening and consent information is entered into the tablet, the new study ID and randomly assigned trial arm will be displayed on screen. Once the personal identifier has been given, study personnel will inform the participant of their assignment and ensuing study procedures, at which time both study personnel and participants are unblinded due to the nature of the study activities.

### Data management

A data management plan will be developed for the study and reviewed by the institutional data protection officer. Source documents are where data are first recorded and from which data to complete participant case report forms (CRFs) will be obtained. These include, but are not limited to, antenatal clinic records (from which physical and clinical examination along with prescribed prophylaxis may be summarised into the CRF), maternal and birth charts (maternal and neonatal health records, infant Road-to-Health cards and admissions register), laboratory and pharmacy records and correspondence.

Clinical forms will be managed using electronic data management systems, with electronic CRFs loaded onto tablets and incorporating predefined range and consistency checks. The Open Data Kit^31^ platform will be used for electronic data collection. This process is Good Clinical Practice (GCP) compliant. Data will be exported from the Open Data Kit server as a comma-separated value file to a local SQL server for data cleaning and processing. Cleaned datasets will be saved to a Microsoft Access database, with regular backups maintained on a secure network-attached storage drive. Any personally identifiable information will be removed from all data files before analysis.

Clinic-level data on the number of women attending antenatal care, screened, eligible, consenting and enrolled by arm will be monitored weekly in the form of a data dashboard. Attrition due to withdrawal, loss to follow-up or other reasons will also be monitored and reasons reported. Weekly reviews will be performed by the data management team to ensure data completeness. Discrepancies will be resolved by triangulating with the ANC records/birth registers, where appropriate.

### Statistical methods

The trial will be reported in accordance with the Consolidated Standards of Reporting Trials (CONSORT) 2025 and Standard Protocol Items: Recommendations for Interventional Trials (SPIRIT) guidelines.^32^ All analyses will be conducted following a pre-specified statistical analysis plan which will be approved by the Trial Steering Committee (TSC) and the Data Safety and Monitoring Board (DSMB). Analyses will be performed by a statistician blinded to trial arm allocation.

The primary analysis will be intention-to-treat. The primary outcome (a composite of preterm birth, LBW and stillbirth) will be compared between trial arms using a generalised linear model with a binomial distribution and log-link function to estimate RRs and corresponding 95% CIs. Models will adjust for clinic. Participants will be excluded from the primary analysis if any component of the composite outcome is missing. Secondary outcomes will be analysed using the same modelling approach as the primary outcome. Each component of the composite primary outcome will also be analysed individually as a secondary outcome. Comparative statistical testing of harms is not planned, and reporting will focus on the frequency and nature of serious adverse events (SAEs), particularly those judged to be related to study procedures or interventions. The primary analysis population (intention-to-treat) will include all randomised participants analysed according to their allocated trial arm, irrespective of adherence to the intervention. Participants will be excluded from the primary analysis if any relevant component of the composite primary outcome (preterm birth, LBW or stillbirth) is missing. Participants who withdraw consent, are lost to follow-up or experience outcomes that preclude completion of postnatal visits will remain in the analysis where outcome data are available. The primary analysis will be repeated using multiple imputation with chained equations to assess the robustness of findings to missing outcome data.

A per-protocol analysis will be conducted as a sensitivity analysis, restricted to participants who received the intervention as specified in the protocol. We will conduct subgroup analyses of the primary composite outcomes and the three individual outcomes of preterm, LBW and stillbirth by the following subgroups: HIV status (positive, negative), gestational age at enrolment (<20 weeks, ≥20 weeks). The proportion of participants testing positive for any STI will be calculated by trial arm. Within the subgroups of participants who test positive at any time during pregnancy for (1) Ct, (2) Ng, (3) Tv or (4) any of the three STIs, the following outcomes will be analysed by arm: the primary composite outcome, preterm birth, LBW and stillbirth. As a secondary analysis we will use the win ratio method for hierarchical composite outcomes. The win ratio has been most widely used in cardiovascular disease and PROMISE will be the first application of it for birth outcomes, to our knowledge.^33^

At its regular meetings, the DSMB will review accruing data on the primary and secondary outcomes by trial arm. If there are emerging trends in the primary outcome in these interim analyses, the Peto-Haybittle stopping rule will be used to determine whether consideration should be given to early stopping of the trial. This conservative stopping rule requires that differences between trial arms in the primary outcome are statistically significant at p<0.001 and preserves the overall type 1 error at α=0.05. Based on its deliberations, the DSMB will advise on whether study modifications and continued recruitment are appropriate.

### Oversight and monitoring

The day-to-day implementation and management of PROMISE will be overseen by the TMG, which will meet in a hybrid fashion on a bi-weekly basis to monitor progress (including recruitment, data quality, intervention delivery and outcome ascertainment). The TMG will ensure that the trial is implemented as planned according to the trial protocol and Manual of Procedures.

A TSC is constituted to provide independent review of the study conduct, progress and findings and review relevant information from other sources (eg, other related trials). The TSC comprises independent experts in clinical trials and statistics, maternal and child health, STIs and health systems. The TSC will review study approvals, progress and obstacles and include statistics of enrolment, refusals to participate/non-enrolment, adverse events, withdrawals and trial efficacy measures. The TSC will also review any SAEs which occur during the study which are highlighted by the Chief Investigator and the DSMB and determine if any of the interventions prove to come at undue risk to the participants.

A DSMB is constituted to provide independent oversight of participant safety and trial conduct, ensuring adherence to the study protocol and consideration of new relevant evidence. The DSMB comprises independent experts in clinical trials, biostatistics and STIs. The DSMB will review SAEs, trial conduct and safety data, providing recommendations to the TSC, sponsor and ethics committees. At its regular meetings, the DSMB will look at all SAEs by study arm to check that there are no unexpected differences. This will not be restricted to those regarded as ‘related’ or ‘unexpected’. The DSMB will consider emerging risks and will advise on whether study modifications, interim analyses or continued recruitment are appropriate. Confidentiality will always be maintained, and a written report summarising the DSMB recommendations will be provided to the investigators, TSC and relevant oversight bodies.

#### Frequency and plans for auditing trial conduct

The trial will be conducted in accordance with the principles of the Declaration of Helsinki and in full conformity with relevant regulations and with the ICH Guidelines for GCP. The trial will be monitored by the LSHTM Research Governance and Integrity Office under their remit as Sponsor, and external trial monitors from the Medical Research Council of Zimbabwe (MRCZ) to ensure adherence to GCP.

### Patient and public involvement

PROMISE will have a Community Advisory Board and will organise structured community engagement activities. The Community Advisory Board will serve as a formal link between the research team and the community. It will provide ongoing input into the conduct of the trial, including advice on framing the study, use of appropriate and sensitive language when addressing topics such as pregnancy loss, and adaptation of community engagement strategies over the course of the trial to remain person-centred and effective. The Community Advisory Board will also support the project team in raising awareness about the study and contributing to our understanding of barriers to early antenatal care attendance.

In addition, patient and public perspectives will be incorporated through the trial’s mixed methods process evaluation. This will include interviews, ethnographic observations and stakeholder workshops involving pregnant women, male partners, community members, traditional healers and traditional birth attendants, healthcare providers and government officials. Findings from these activities will be used iteratively to inform implementation and to understand acceptability, feasibility and context relevant to intervention delivery and potential scale-up. Lastly, participant and community representatives will also be involved in dissemination activities. Trial results will be fed back through community dissemination workshops, Community Advisory Board engagement and community-tailored outputs developed with input from clinic staff and the Community Advisory Board, ensuring that findings are accessible and relevant to participating communities.

### Ethics and dissemination

#### Research ethics approval and consent to participate

The trial was approved by the Medical Research Council of Zimbabwe (MRCZ/A/3289), the Biomedical Research and Training Institute Institutional Review Board (AP204), the Joint Research and Ethics Committee (JREC/380/2025), the Sally Mugabe Central Hospital Ethics Committee (SMCHEC070425/58) and the London School of Hygiene and Tropical Medicine ethics committee (31428). All participants will provide written informed consent to participate. Pregnant mothers under the age of 18 years are considered emancipated. Every protocol amendment will be approved by the LSHTM ethics board and MRCZ. Once approved, trial registration on PACTR will be updated to reflect the changes made in the protocol amendments. All protocol amendments will be approved by the TSC and the DSMB prior to ethics submission.

#### Harms

Trial activities undertaken in the PROMISE trial pose minimal risk to participants. Collection of vaginal swabs may cause brief discomfort but should not result in physical harm to the mother or baby. Participants may have adverse reactions to antibiotics administered following a positive STI result, and they will hence be monitored following treatment. Psychological or social harms may occur following disclosure of an STI/HIV diagnosis, including stress, stigma or intimate partner violence. There is also a potential risk of loss of privacy or confidentiality.

Adverse events will be monitored throughout the trial. SAEs are defined in accordance with the principles of International Council for Harmonisation Good Clinical Practice. The severity of adverse reactions will be graded using the Common Terminology Criteria for Adverse Events. SAEs will be recorded on regulatory reporting forms and reported immediately (within 24 hours of awareness) to the Trial Coordinator and reviewed by the Chief Investigator, who will assess seriousness, relatedness and unexpectedness. SAEs will be reviewed by the DSMB in real time. SAEs deemed related or unexpected will be reported in an expedited manner to the relevant ethics committees and regulatory authorities, in line with local and international guidelines.

Additional care required for conditions identified through study procedures will be facilitated in line with routine services. This will include treatment and referral for HIV, syphilis, hypertensive disorders of pregnancy, anaemia and other medical or obstetric conditions identified during visits. Women newly diagnosed with HIV will be linked to HIV care and antiretroviral therapy through clinic services, and infants born to mothers living with HIV or exposed to syphilis will receive prophylaxis and management according to national guidelines. These services will all be provided through the clinic though PROMISE will support referral where needed. Participants experiencing psychosocial distress related to STI or HIV diagnoses, pregnancy complications or adverse birth outcomes will be offered counselling and referral to support services, including referral to SALT Africa (Someone Always Listens to You), a local maternal mental health specialist organisation based at a national tertiary hospital in Zimbabwe. The intervention will consist of a weekly support package, delivered via telephone or in-person to ensure continuity of care and participant accessibility and tailored to the participant’s preference.

There will be no trial-specific provisions for continued provision of STI screening after trial completion. Following completion of study participation, women and infants will continue to access care through routine services. However, throughout the trial, PROMISE will engage with district-level, national, regional and international policy makers to discuss the systems requirements for implementation at scale, if shown to be successful. The trial sponsor will have insurance in place to cover non-negligent harm arising from trial participation, in line with sponsor and regulatory requirements. Participants who suffer harm as a result of study participation will be eligible for compensation through these mechanisms. Clinical management of adverse events will be provided promptly and free of charge through established pathways.

### Confidentiality

All documents will be stored safely in confidential conditions and held for 5 years after the end of the trial (8 weeks after last participant has delivered) in accordance with GCP principles. On all trial-specific documents, other than the signed consent forms, the participant will be referred to by the trial participant number (study ID) not by name. Samples sent to the laboratory will be identified through participant-specific barcodes which will be linked to the study ID and not to any identifying information.

### Dissemination

The dissemination strategy is designed to communicate effectively with a broad range of target audiences including trial communities, other researchers, ministries of health and wider society. A study-specific website will be designed to share trial progress and results.

In addition to disseminating trial results at international conferences and through peer reviewed manuscripts and policy briefs, social media platforms (eg, LinkedIn) and newsletters will be employed to communicate progress of the project. Brochures outlining the aims of the project and site-specific findings will be co-developed with the TMG, clinic staff and the Community Advisory Board. Regular engagement with the Community Advisory Board will be maintained to provide updates and share results. Community and national dissemination workshops will be organised to feed results back. In addition, biannual stakeholder meetings will be convened to engage national, regional and international key stakeholders in the trial’s design, implementation and results throughout the trial.

Publication of the results of this study will be governed by the requirements of the UK Research and Innovation open access policy. All publications will be made available through PubMed Central and Europe PubMed Central as soon as possible and within 6 months of the journal publisher’s official date of final publication. Where possible (and when an open access fee is paid), research papers will be licenced with a Creative Commons Attribution licence (CC-BY). Where feasible, key findings from research papers will be explained in accompanying community-tailored outputs, for example brief animated films. In order to ensure data confidentiality, participants will be identified only by their unique study ID. All data will be anonymised so that no link to name or other identifiable information will be stored.
