# Supplementary material for: Protecting mothers and their infants through screening and comprehensive management of sexually transmitted infections in antenatal care in Zimbabwe (PROMISE): study protocol for an open label individually randomised controlled trial
Source: BMJ Open. 2026 Oct 15;16(10):e126249. doi: 10.1136/bmjopen-2026-126249 (PMC13691885; doi:10.1136/bmjopen-2026-126249)
Supplement: Supplementary file 1 [file bmjopen-2026-126249.reviewer_comments.pdf]

PEER REVIEW HISTORY

BMJ Open publishes all reviews undertaken for accepted manuscripts. Reviewers are asked to complete a checklist review form and are provided with free text boxes to elaborate on their assessment. These free text comments are reproduced below.

ARTICLE DETAILS

Title (Provisional)

Protecting mothers and their infants through screening and comprehensive management of sexually transmitted infections in antenatal care in Zimbabwe (PROMISE): study protocol for an open label individually randomised controlled trial

Authors

Larsson, Leyla; Kabongo, Jacqueline; Murewanhema, Grant; Simms, Victoria; Dauya, Ethel; Bandason , Tsitsi; Mackworth-Young, Constance R.S.; Bozzani, Fiammetta Maria; Butcher, Robert; Machiha, Anna; Low, Nicola; Sturgeon, Jonathan Peter; Blencowe, Hannah; Dziva Chikwari, Chido; Stranix-Chibanda, Lynda; Juru, Agnes; Sisya, Lucia; Bernays , Sarah; Kranzer, Katharina; Marks, Michael; Ferrand, Rashida A.; Trial Consortium, PROMISE

This article was not externally reviewed at BMJ Open. Protocols that have been independently assessed prior to submission to BMJ Open are usually fast-tracked to publication on the grounds that further substantial changes will not be possible. This independent assessment will usually be external, independent review for both a substantial grant award from a non-commercial or government funder and ethics approval.
